# 

**Published:** 1929

**Authors:** 


### The Medical Library of the University of Bristol,

WITH WHICH IS INCORPORATED

**The Library of the Bristol Medico-Chirurgical Society.**

---

*The following donations have been received since the publication of the list in October,* 1929.

December, 1929.

| | | |
|---|---|---|
| Professor R. J. A. Berry (1) | .. .. .. | 26 volumes |
| Professor E. W. Hey Groves (2) | .. .. | 38 ,, |
| Mrs. E. G. D. Hailes (3) | .. .. .. | 1 volume |
| Government of India (4) | .. .. .. | 1 ,, |
| Rockefeller Foundation (5) | .. .. .. | 1 ,, |
| Mr. Rendle Short, F.R.C.S. (6) | .. .. | 2 volumes |

Unbound periodicals have also been received from Professor E. W. Hey Groves and Dr. Carey Coombs.

### THE ONE HUNDRED AND THIRTIETH LIST OF BOOKS.

The figures in round brackets refer to the figures after the names of the donors. The books to which no such figures are attached have either been bought from the Library Fund or received through the *Journal.*

| | | | | |
|---|---|---|---|---|
| **Back, I., and Edwards, A. T.** | *Synopsis of Surgery* | | (2) | 1921 |
| **Ballance, Sir C. A.** | *History of Surgery of Brain* | | (2) | 1922 |
| **Barker, L. F.** | *Laboratory Manual of Human Anatomy* | | (1) | 1904 |
| **Benisty, A.** | *Clinical Forms of Nerve Lesions* | | (2) | 1918 |
| **Benisty, A.** | *Treatment of Nerve Lesions* | | (2) | 1918 |
| **Berry, R. J. A.** | *Essentials of Regional Anatomy* | | (1) | 1900 |
| **Berry, R. J. A.** | *Various Papers by* | | (1) | 1898, etc. |
| **Billington, W.** | *Movable Kidney* | 2nd Ed. | | 1929 |
| **Böhler, L.** | *Treatment of Fractures* | | | 1929 |
| **Broca, A.** | *After-effects of Wounds of Bones and Joints* | | (2) | 1918 |
| **Broca, A., and Ducroquet** | *Artificial Limbs* | | (2) | 1918 |
| **Chatterji, K. K.** | *Handbook of Operative Surgery* | 2nd Ed. | (2) | 1921 |
| **Cotterell, E.** | *The Pocket Gray's Anatomy* | | (2) | 1899 |
| **Crile, G. W.** | *The Kinetic Drive* | | (2) | 1916 |
| **Crile, G. W., and Lower, W. E.** | *Anoci-Association* | | (2) | 1914 |
| **Cumston, C. G., and Patry, G.** | *Surgical Treatment of Non-malignant Affections of Stomach* | | (2) | 1921 |
| **Cunningham** | *Text-book of Anatomy* | | (1) | 1902 |
| **Danforth, C. H.** | *Hair* | | (1) | 1925 |
| **Davis, J. S.** | *Plastic Surgery* | | (2) | 1919 |
| **Dercum, F. X.** | *Physiology of Mind* | 2nd Ed. | (1) | 1929 |
| **Digby, K. H.** | *Immunity in Health* | | (1) | 1919 |
| **Dollar, A. W.** | *Regional Veterinary Surgery* | | (2) | 1912 |

| | | | | |
|---|---|---|---|---|
| **Doran, A. H. G.** | *Catalogue of Obstetrical Instruments of Museum of R.C.S.*, Part I. | | (2) | 1921 |
| **Douthwaite, A. H.** | *Treatment of Rheumatoid Arthritis* | | | 1929 |
| **Dujarier, C. L.** | *Anatomie des Membres* | | (1) | 1906 |
| **Ernst, F. G.** | *Orthopaedic Apparatus* | | (2) | 1921 |
| **Elder, W.** | *Studies in Psychology* | | (1) | 1927 |
| **Falconer, W.** | *Essay on the Bath Waters* | | | 1770 |
| *Guide to Exhibition Galleries of B.M.* | | | (2) | 1896 |
| **Herman** | *Difficult Labour* | 7th Ed. | | 1929 |
| **Higgins, H.** | *Biological Reversion and Hippocratic Anatomy* | | | 1929 |
| **Howson** | *Handbook for the Limbless* | | (2) | 1916 |
| **Hull, A. G.** | *Surgery in War* | | (2) | 1916 |
| **Jones, R.** | *Notes on Military Orthopedics* | | (2) | 1917 |
| **Jeanneney, G.** | *Cancer* | | | 1929 |
| **Keen, W. W.** | *Treatment of War Wounds* | 2nd Ed. | (2) | 1918 |
| **Kemper, G. W. H.** | *The World's Anatomists* | | (1) | 1905 |
| **Kirk, N. T.** | *Amputations* | | (2) | 1924 |
| **Le Double, A. F.** | *Traité des Variations du Système Musculaire de l'Homme.* Vols. I., II. | | (1) | 1897 |
| **Lipschütz, A.** | *Internal Secretions of Sex Glands* | | | 1924 |
| **Mackie, T. G.** | *Post-operative Tetanus* | | | 1928 |
| **Magnus, G.** | *Frakturen und Luxationen* | | (2) | 1923 |
| **Martinier, P. and Lemerle, G.** | *Injuries of Face and Jaw* | | (2) | 1917 |
| **Mennell, J. B.** | *Massage* | | (2) | 1917 |
| **Merkel, F.** | *Handbuch der Topographischen Anatomie.* Vol. I., III. | | (1) | 1899 |
| **Monrad-Krohn** | *Clinical Examination of Nervous System* | 4th Ed. | | 1928 |
| **Norris, G. W.** | *Blood Pressure* | 3rd Ed. | (2) | 1917 |
| *Notes on Cancer* | | | (2) | n. d. |
| **Paget, S.** | *Pasteur and after Pasteur* | | (2) | 1914 |
| **Paterson, A. M.** | *The Sternum* | | (1) | 1904 |
| **Paterson, H. J.** | *Indigestion* | | | 1929 |
| **Playfair, J.** | *Method of Constructing Vapor Baths* | | | 1783 |
| **Power, D'Arcy** | *The Practitioner's Surgery* Vol. I. | | (2) | 1919 |
| *Practitioner's Guide to Clinical Research* | | 3rd Ed. | (2) | 1928 |
| **Rawlings, L. B.** | *Landmarks of the Human Body* | 7th Ed. | | 1929 |
| **Roberts, H.** | *Recent Work on Colporrhaphy* | | | 1929 |
| **Savage, W. G.** | *Prevention of Tuberculosis* | | | 1929 |
| **Schmidt, O.** | *The Mammalia* | | (1) | 1885 |
| **Shennan, T.** | *Post-mortems and Morbid Anatomy* | | | 1927 |
| **Sonntag, E.** | *Grundriss der gesamten Chirurgie* | 2nd Ed. | (2) | 1923 |
| **Sternberg, W.** | *Technique and Method of use of Sternberg's Gastroscopy* | | | 1929 |
| **Stopes, M. C.** | *Wise Parenthood* | 5th Ed. | (2) | 1919 |
| **Souttar, H. S.** | *Radium and its Surgical Applications* | | | 1929 |
| **Syme, J.** | *Diseases of the Rectum* | 3rd Ed. | (1) | 1854 |
| **Testut, L.** | *Traité d'anatomie Humaine.* Vol, I. | 3rd Ed. | (1) | 1896 |
| **Thompson, A., and Miles, A.** | *Manual of Surgery* Vol. II. | 6th Ed. | (2) | 1921 |
| **Titchener, E. B.** | *An Outline of Psychology* | | (2) | 1897 |
| **Triepel, H.** | *Nomina Anatomia* | | (1) | 1910 |

**Triepel, H.** .. .. .. *Die Anatomischen Namen* .. .. 3rd Ed. (1) 1910
**Tubby, A. H.** .. .. *Malleable Iron Splints* .. .. .. .. .. .. (2) 1915
**Turner, A. L., and Porter, W. G.** *Skiagraphy of the Accessory Nasal Sinuses* .. .. .. .. .. .. .. .. .. .. (1) 1912
**Vazifdar, N. T.** .. .. *Physiology of the Central Nervous System* 5th Ed. (6) 1929
**Whetham, W. C. D., and Whetham, C. D.** *An Introduction to Eugenics* (1) 1912
**Wiggers, C. T.** .. .. *The Pressure Pulses in the Cardiovascular System* .. .. .. .. .. .. .. .. .. .. (6) 1928

TRANSACTIONS, REPORTS, JOURNALS, ETC.

Medical Society's Transactions. Vol. 52.. .. .. .. .. .. .. .. .. .. 1929
Methods and Problems of Medical Education. 14th Series .. .. (5) 1929
Ophthalmic Yearbook. 7th .. .. .. .. .. .. .. .. .. .. .. .. (3) 1910
Progressive Medicine. Sept., 1929 .. .. .. .. .. .. .. .. .. .. .. 1929
Publications of First Orthopedic Division of the Hospital for Ruptured and Crippled. Vol. I. .. .. .. .. .. .. .. .. .. .. .. .. .. (2) 1922
Reports of St. Bartholomew's Hospital. Vol. 62 .. .. .. .. .. .. .. 1929
Surgical Clinics of North America. Oct., 1929 .. .. .. .. .. .. .. 1929
Transactions of the 7th Congress of Far Eastern Association of Tropical Medicine. Vol. 2. 1927 .. .. .. .. .. .. .. .. .. .. .. .. (4) 1929
Westminster Hospital Reports. Vol. 19 (2); Vol. 21 .. .. 1924 and 1929
Zentralblatt für die gesamten Chirurgie. Bde. I., II. .. .. .. (2) 1913

### Local Medical Notes.

**Harveian Society: Buckston Browne Prize.**—The President and Council of this Society have awarded the biennial Buckston Browne Prize of £100 and medal to Dr. C. Bruce Perry for an essay on "Chronic Streptococcal Illnesses."

NEW APPOINTMENTS.

**University of Bristol.**—Demonstrator of Anatomy: C. B. Perry, M.D., M.R.C.P.

**Bristol General Hospital.**—Surgeon: Duncan Wood, F.R.C.S. Dental Surgeon: Norman H. Kettlewell, L.D.S., R.C.S. (Eng.), H.D.D., R.C.S. (Edin.), L.M.S.S.A. (Lond.). Assistant Physician to the Skin Department: Norman F. C. Burgess, B.A., M.D., M.R.C.P. Assistant Surgeon: S. J. H. Griffiths, M.B., Ch.B., F.R.C.S.

**Bristol Royal Infirmary.**—Senior Resident Officer: A. R. Williams, M.B., Ch.B. (Bristol).

EXAMINATION RESULTS.

**University of Bristol.**—Students of the University have recently passed the following examinations :—

M.B., CH.B.—*Supplementary First Examination. Pass :* Grace J. V. Ball, K. P. D. Griffiths, G. L. L. Gurney, T. R. V.

Gurney, K. C. P. Smith, S. P. N. Williams. *In Chemistry and Biology only :* Irene G. Hamilton, Ursula W. Wood.

L.D.S.—*Supplementary Preliminary Science Examination. Pass : In Biology* (*Completing Examination*), R. L. Royal.

**University of London.**—M.B., B.S. : Doreen G. C. Nixon, L. E. Vine. Royal College of Surgeons.—Final F.R.C.S. : E. F. King. Primary F.R.C.S. : T. H. Berrill, J. T. Chesterman.

CONJOINT BOARD. — M.R.C.S., L.R.C.P. — *Pass : In Elementary Biology,* E. R. Atchison. *In Medicine,* C. H. Pauli, *A. C. Price. *In Surgery,* *A. C. Price, G. F. Langley. *In Midwifery,* *A. C. Price, G. F. Langley, W. Woolley, W. L. Sleight, A. A. G. Flemming, *N. L. Price. *In Pathology,* K. I. Nicholls.

L.D.S., R.C.S.—*Part I.* (*Dental Mechanics*). *Pass :* R. E. Morgan, W. A. Duly, W. U. Harwood. *Part II.* (*Dental Metallurgy*). *Pass :* R. E. Morgan, T. M. Ayliffe, E. R. Dowland. *Part III.* (*a*) (*Anatomy and Physiology*). *Pass :* F. S. Bromley, R. H. Green, O. F. Harley, M. D. Hooper, P. J. F. Shepherd, H. W. South, W. N. Trays. *Part III.* (*b*) (*Dental Anatomy and Physiology*). *Pass :* G. E. Chadd.

**Society of Apothecaries.** — L.M.S.S.A. (October, 1929). : *T. M. White.

* Qualified.

**The Annual Dinner** of the Bristol Medical School was held at the University Union (Victoria Rooms) on Thursday, December 12th. Professor G. A. Buckmaster was in the Chair, and the guest of the evening was Sir John Rose Bradford, K.C.M.G., M.D., F.R.S., President of the Royal College of Physicians.

After the king's health had been honoured, the President proposed the toast of "the Guest of the Evening," which was received with great enthusiasm. Sir John Rose Bradford returned thanks, and in his reply proposed the health of "The Bristol Medical School." Mr. A. E. Iles, F.R.C.S., and Mr. J. L. S. Coulter responded for the past and present members of the School. Dr. J. A. Nixon proposed the toast of the President, and Professor Buckmaster replied.

After the tables had been cleared the usual dance took place, arranged, as in past years, by the wives of the staffs. When the gathering broke up at midnight the general impression was that an exceptionally pleasant evening had come to an end.

### Index.

Adams, A. W.—The Rôle of Pyelography in Renal Disorders, 49.
Adenoma, Renal.—G. Hadfield, 211.
Anæsthetics.—S. V. Stock, 197.
Angioma of the Cerebellum, Polycystic Pancreas and Renal Adenoma (Lindau's Syndrome), The Association between.—G. Hadfield, 211.
Aortic Wall, Histology of, in Acute Rheumatism.—J. J. Giraldi, 145.
Appointments—
 Claremont, L. E.—Director of Eastman Clinic, London, 241.
 Cooke, R. V.—Junior Assistant to Surgical Unit of National School of Medicine, Cardiff, 242.
 Coombs, C. F.—Lumleian Lecturer, 1930, 242.
 Groves, E. W. Hey.—Hunterian Orator, 1930, 100.
 Mackie, F. P.—Honorary Surgeon to the King, 99.
 Tratman, E. K.—Professor of Dental Surgery, University of Singapore, 241.
Arterial Changes, Senile, in a Child of Seven Weeks.—F. W. T. Hughes and C. B. Perry, 219.
Arterial Tension, High.—C. F. Coombs, 35.
Association between Angioma of the Cerebellum, Polycystic Pancreas and Renal Adenoma (Lindau's Syndrome). G. Hadfield, 211.
Aubrey, T., and Britton, R. B.—An Epidemic of Virulent Diphtheria, 141.

Bodman, F., and Taylor, A. L.—Portal Pyæmia following Diverticulitis, 131.
Books, Reviews of—
 Abel, A.L.—Œsophageal Obstruction, 309.
 Alcohol and Human Life.—C. C. Weeks, 224.
 Alport, A. C.—Nephritis, 80.
 Artificial Sunlight and its Therapeutic Uses.—F. H. Humphris, 307.
 Bennett, T. I.—Nephritis, 164.
 Billington, W.—Movable Kidney, 308.
 Blondel, C.—The Troubled Conscience, 81.
Books, Reviews of (continued)—
 Blood Pressure, Low.—J. F. Halls Dally, 79.
 Böhler, Lorenz.—The Treatment of Fractures, 307.
 Bourne, G., and Stone, K.—Principles of Clinical Pathology in Practice, 86.
 Cade, S., and Paterson, D.—Westminster Hospital Reports, Vol. 20, 302.
 Cameron, H. C.—The Nervous Child, 305.
 Cancer of the Uterus, The Radium Treatment of.—The Cancer Research Committee of the Marie Curie Clinic, 305.
 Cancer Research Committee of the Marie Curie Clinic, The.—The Radium Treatment of Cancer of the Uterus, 305.
 Cancer.—G. Jeanneney, 306.
 Carling, E. Rock.—Westminster Hospital Reports. Vol. 21. Radium Practice, 302.
 Cassie, E.—Maternity and Child Welfare, 85.
 Chagas, C.—"Terminal" Disinfection, 164.
 Chandler, F. G.—Lipoidol in the Diagnosis of Thoracic Disease, 87.
 Chiropody.—E. G. V. Runting, 87.
 Cope, V. Z.—Some Principles of Minor Surgery, 224.
 Critchley, M.—Mirror Writing, 78.
 Curschmann, Hans.—Endocrine Disorders, 299.
 Cytoarchitectonics.—C. von Economo, 78.
 Deafness and its Alleviation.—V. Nesfield, 86.
 Diabetes, A Patient's Manual of.—H. W. Moxon, 85.
 Differential Diagnosis.—H. French, 87.
 Difficult Labour.—G. E. Hermann, 304.
 Douthwaite, A. H.—Injection Treatment of Varicose Veins, 3rd Ed., 83; The Treatment of Rheumatoid Arthritis, 300.
 Economo, C. von.—Cytoarchitectonics of the Human Cerebral Cortex, 78.

BOOKS, REVIEWS OF (continued)—
Endocrine Disorders.— Hans Curschmann, 299.
Essays, Medical.—C. O. Hawthorne, 77.
Forrester-Brown, M. F.—Deformities in Infancy and Childhood, 88.
Fractures and Dislocations in General Practice, The Treatment of.—C. M. Page and W. R. Bristowe, 308.
Fractures, The Treatment of.—Lorenz Böhler, 307.
French, H.—An Index of Differential Diagnosis, 87.
Gastroscopy, Technique of. — W. Sternberg, 225.
Gillespie, R. D.—Hypochondria, 306.
Gordon, R. G.—Autolycus, 80.
Gould, Sir A. Pearce.—Elements of Surgical Diagnosis, 7th Ed., 83.
Halls-Dally, J. F. — Low Blood Pressure, 79.
Hawthorne, C. O.—Medical Essays, 77.
Hemorrhoids.—A. S. Morley, 226.
Hermann, G. E.—Difficult Labour, 304.
Human Tuberculosis of Bovine Origin, The Prevention of.—W. G. Savage, 303.
Humphris, F. H.—Artificial Sunlight and its Therapeutic Uses, 307.
Hygiene, Naval.—T. B. Shaw, 301.
Hygiene and Public Health.—Parkes and Kenwood, 165.
Hypochondria.—R. D. Gillespie, 306.
Indigestion.—H. J. Paterson, 301.
Infancy, Deformities in.—M. F. Forrester-Brown, 88.
Infectious Diseases.—Ker, 223.
Jeanneney, G.—Cancer, 306.
Jones, D. W. C.—Elementary Medicine in Terms of Physiology, 81.
Ker's Infectious Diseases, 223.
Landmarks and Surface Markings of the Human Body.—L. B. Rawling, 299.
Lord, J. R. — Contributions to Psychiatry, Neurology and Sociology, 223.
McCurrich, H. J.—The Treatment of the Sick Poor of this Country, 163.
Manson.—Tropical Diseases, 165.
Maternity and Child Welfare.—E. Cassie, 85.
Medicine, Elementary.—D. W. C. Jones, 81.
Monrad-Krohn, G. H.—The Clinical Examination of the Nervous System, 300.

BOOKS, REVIEWS OF (continued)—
Moore, I.—The Tonsils and Adenoids, 88.
Morley, A. S.—Hemorrhoids, 226.
Mott Memorial, 223.
Movable Kidney.—W. Billington, 308.
Moxon, H. W.—A Patient's Manual of Diabetes, 85.
Nægeli, Th.—A Graphic Guide to Elementary Surgery, 226.
Naval Hygiene. — Thomas Brown Shaw, 301.
Nephritis.—A. C. Alport, 80.
Nephritis.—T. I. Bennett, 164.
Nervous System, The Clinical Examination of.—G. H. Monrad-Krohn, 300.
Nervous Child, The.—H. C. Cameron, 305.
Nesfield, V.—Deafness and its Alleviation, 86.
Obstetrics.—Tweedy, 163.
Œsophageal Obstruction. — A. L. Abel, 309.
Page, C. M., and Bristowe, W. R.—The Treatment of Fractures and Dislocations in General Practice, 308.
Parkes and Kenwood.—Hygiene and Public Health, 165.
Paterson, H. J.—Indigestion, 301.
Pathology, Clinical.—G. Bourne and K. Stone, 86.
Physical Treatment.—M. B. Ray, 225.
Practice of Medicine, A Text-book of the.—Frederick W. Price, 300.
Price, Frederick W.—A Text-book of the Practice of Medicine, 300.
Psychiatry, Neurology and Sociology, Contributions to.—J. R. Lord, 223.
Radium and its Surgical Application. —H. S. Souttar, 303.
Rawling, L. B.—Landmarks and Surface Markings of the Human Body, 299.
Ray, M. B.—On Prescribing Physical Treatment, 225.
Rheumatoid Arthritis, The Treatment of.—A. H. Douthwaite, 300.
Runting, E. G. V.—Practical Chiropody, 3rd Ed., 87.
Savage, W. G.—The Prevention of Human Tuberculosis of Bovine Origin, 303.
Shaw, T. B.—Naval Hygiene, 301.
Souttar, H. S.—The Art of Surgery, 82; Radium and its Surgical Application, 303.
Sternberg, W.—Technique of Gastroscopy, 225.

BOOKS, REVIEWS OF (continued)—
Surgery, Art of.—H. S. Souttar, 82.
Surgery, Elementary.—Nægeli, Th., 226.
Surgery, Minor.—V. Z. Cope, 224.
Surgical Diagnosis.—Sir A. Pearce Gould, 83.
Symptomatology, Index of.—H. L. Tidy, 84.
Thoracic Diseases.—F. G. Chandler, 81.
Tidy, H. L.—An Index of Symptomatology, 84.
Tonsils and Adenoids.—I. Moore, 88.
Tropical Diseases.—Manson, 165.
Tweedy.—Practical Obstetrics, 6th Ed., 163.
Varicose Veins.—A. H. Douthwaite, 83.
Ward, E.—Medical Adventure, 77.
Weeks, C. C.—Alcohol and Human Life, 224.
Westminster Hospital Reports. Vol. 20—S. Cade and D. Paterson, 302.
Westminster Hospital Reports. Vol. 21. Radium Practice.—E. R. Carling, 302.

Borderland between Surgery and Gynæcology, The.—E. W. Hey Groves, 185.
Bristol Medical School, 232.
Bristol Medico-Chirurgical Society, 91, 169, 313; Annual Dinner, 233.
British Medical Association.—Bath and Bristol Branch, 90, 315.
Britton, R. B., and Aubrey, T.—An Epidemic of Virulent Diphtheria, 141.

Carleton, H. H.—Significance of Sleep Disorders, 173.
Case, A, of Primary Sarcoma of the Spleen.—A. L. Taylor, 121.
Case, A, of Rectal Prolapse.—D. Robertson and G. D. C. Tasker, 71.
Cerebellum, Angioma of the.—G. Hadfield, 211.
Clinical Diagnosis of Primary Syphilis.—A. M. Critchley, 127.
Clinical Society of Bath, 94.
Coombs, C. F.—The Distal Phenomena that accompany High Arterial Tension, 35.
Critchley, A. M.—Clinical Diagnosis of Primary Syphilis, 127.

Daukes, S. H.—The Wellcome Museum of Medical Science, 236.
Diphtheria, Virulent, An Epidemic of.—T. Aubrey and R. B. Britton, 141.
Distal Phenomena, The, that accompany High Arterial Tension.—C. F. Coombs, 35.
Diverticulitis, Portal Pyæmia following.—F. Bodman and A. L. Taylor, 131.

Eastman Dental Clinic, London, 234.
Editorial Notes, 90, 167, 231, 310.
Epidemic, An, of Virulent Diphtheria.—T. Aubrey and R. B. Britton, 141.

Foreign Bodies in the Gullet.—E. Watson-Williams, 109.

Giraldi, J. J.—Histology of the Aortic Wall in Acute Rheumatism, 145.
Goitre, Parenchymatous, Treatment of.—W. A. Jackman, 277.
Gordon, R. G.—The Early Treatment and Diagnosis of Anterior Poliomyelitis, 289.
Groves, E. W. Hey.—The Borderland between Surgery and Gynæcology, 185.
Gullet, Foreign Bodies in.—E. Watson-Williams, 109.
Gynæcology and Surgery, The Borderland between.—E. W. Hey Groves, 185.

Harvey, William, and Kingsweston, 90.
Heimann, H. L.—Three Cases of Sino-auricular block, 285.
High Arterial Tension.—C. F. Coombs, 35.
Histology, The, of the Aortic Wall in Acute Rheumatism.—J. J. Giraldi, 145.
Hughes, F. W. T., and Perry, C. B.—Senile Arterial Changes in a Child of Seven Weeks, 219.

Jackman, W. A.—The Treatment of Parenchymatous Goitre, 277.

Library, Medical, of the University of Bristol, 97, 170, 240, 335.
Lindau's Syndrome, 211.
Local Medical Notes, 99, 171, 241, 337.
Long Fox Memorial Lecture, The.—Ten Years' Progress in Surgical Treatment, A. R. Short, 243.

Management, The, of the Normal Puerperum.—H. J. Drew Smythe, 59.
Medical Library of the University of Bristol, 97, 170, 240, 335.
Mental Defectives, Care and Treatment of.—H. L. Ormerod, 1.

Obituary—
H. G. Eycott, 168.
C. A. Morton, 238.
Ormerod, H. L.—Some Points on the Care and Treatment of Mental Defectives, 1.

Pancreas, Polycystic.—G. Hadfield, 211.
Perry, C. B., and Hughes, F. W. T.—Senile Arterial Changes in a Child of Seven Weeks, 219.
Poliomyelitis, Anterior, The Early Treatment and Diagnosis of.—R. G. Gordon, 289.
Portal Pyæmia following Diverticulitis. —F. Bodman and A. L. Taylor, 131.
Puerperum, The Management of the Normal.—H. J. Drew Symthe, 59.
Pyæmia, Portal, following Diverticulitis. F. Bodman and A. L. Taylor, 131.
Pyelography in Renal Disorders.—A. W. Adams, 49.

Radium and its Surgical Applications.—H. S. Souttar, 19.
Rectal Prolapse, A Case of.—D. Robertson and G. D. C. Tasker, 71.
Renal Adenoma.—G. Hadfield, 211.
Renal Disorders, The Rôle of Pyelography in.—A. W. Adams, 49.
Rheumatism, Histology of Aortic Wall in.—J. J. Giraldi, 145.
Robertson, D., and Tasker, D. G. C.—A Case of Rectal Prolapse, 71.
Rôle, The, of Pyelography in Renal Disorders.—A. W. Adams, 49.

Sarcoma of the Spleen.—A. L. Taylor 121.
Senile Arterial Changes in a Child of Seven Weeks.—F. W. T. Hughes and C. B. Perry, 219.
Short, A. R.—Ten Years' Progress in Surgical Treatment, 243.
Significance of Sleep Disorders.—H. H. Carleton, 173.
Sino-Auricular Block, Three Cases of.—H. L. Heimann, 285.
Sleep Disorders, Significance of.—H. H. Carleton, 173.
Smythe, H. J. Drew.—The Management of the Normal Puerperum.—59.
Societies—
Bristol Medico-Chirurgical Society, 91, 169, 233, 313.
British Medical Association.—Bristol and Bath Branch, 90, 315.
Clinical Society of Bath, 94.
Some Points on the Care and Treatment of Mental Defectives. — H. L. Ormerod, 1.
Souttar, H. S.—Radium and its Surgical Applications, 19.
Spleen, Case of Primary Sarcoma of.—A. L. Taylor, 121.
Stock, C. V.—Anæsthetics, 197.
Surgery and Gynæcology, The Borderland between.—E. W. Hey Groves, 185.
Surgical Treatment, Ten Years' Progress in.—A. R. Short, 243.
Syphilis, Clinical Diagnosis of.—A. M. Critchley, 127.

Tasker, D. G. C., and Robertson, D.—A Case of Rectal Prolapse, 71.
Taylor, A. L.—A Case of Primary Sarcoma of the Spleen, 121.
Taylor, A. L., and Bodman, F.—Portal Pyæmia following Diverticulitis, 131.

Watson-Williams, E.—Foreign Bodies in the Gullet, 109.
Wellcome Medical Museum of Medical Science.—S. H. Daukes, 236.

J. W. ARROWSMITH LTD., BRISTOL.

